# "Diabetic" Gangrene

**Published:** 1899-12-30

**Authors:** 


### "DIABETIC" GANGRENE.

It has long been recognised not only that there is an association between glycosuria and the occurrence of gangrene, but that gangrene occurring in this connection is very apt to lead to a fatal issue. Thus it has happened that for a long time diabetic gangrene has been regarded as something apart from other forms, and as hardly admitting of surgical intervention. In fact, it is only recently that much hope of success from surgical interference in such cases has been offered. In a very interesting paper on the subject, however, Mr. Cuthbert Wallace[1] shows good cause for reconsidering many of the views which have long been current on the subject, and for believing on the one hand that gangrene occurring in a patient suffering from glycosuria is produced by vascular changes in just the same way as in patients whose urine is free from sugar; and, on the other hand, that in some cases the gangrene and the resulting toxæmia may be the actual cause of the glycosuria which is discovered, while in others the gangrene may have aggravated a pre-existing glycosuria. This puts the question of operation in quite a different light. By far the most constant pathological condition met with in cases of "diabetic" gangrene is, he says, arterial degeneration, and it is to this that we must look for the causation of the disease. This may possibly in some cases arise as a direct result of the glycosuria; but it must be remembered that gout and kidney disease, both of which may cause arterial changes, often accompany diabetes, while endarteritis obliterans and syphilitic arteritis must also be excluded before diabetes can be pronounced to be the cause. Taking all these things into consideration, the conclusion is arrived at that the best chance of recovery in cases of diabetic gangrene is offered by removal of the limb near the trunk; that this measure should be undertaken before the patient is reduced by septic absorption; and that the presence of glycosuria may be an indication, instead of a contra-indication, for operation.

[1] Lancet, Dec. 23.

